# Effect of temperature on the setting time of Mineral Trioxide Aggregate (MTA)

**Published:** 2015

**Authors:** R Sharifi, A Araghid, S Ghanem, A Fatahi

**Affiliations:** * Endodontics Department, School of Dentistry, Kermanshah University of Medical Sciences, Kermanshah, Iran; ** School of Dentistry, Kermanshah University of Medical Sciences, Kermanshah, Iran; *** Medical Biology Research Center, School of Dentistry, Kermanshah University of Medical Sciences, Kermanshah, Iran

**Keywords:** Mineral Trioxide Aggregate (MTA), setting time, temperature

## Abstract

**Introduction:** Mineral trioxide aggregate (MTA) has numerous applications in dentistry due to various advantages. However, its long setting time has still remained a problem. The current study was conducted to investigate the effect of temperature (ambient and distilled water temperature) on the setting time of mineral trioxide aggregate (MTA).

**Materials and methods:** This experimental study comprised of two parts. In the first part, MTA and distilled water samples were kept at ambient temperature for 24 hours (before mixing: effect of distilled water temperature on the setting time of MTA and after mixing: effect of distilled water and ambient temperature on the setting time of MTA), and analyzed and divided into three groups: group 1 (4°C), group 2 (37°C) and group 3 (90°C). The mixed samples were placed in the glass cylinders with an internal diameter of 8 mm and a height of 10 mm, and kept at 37°C temperature and 100% humidity. In the second part, the samples were prepared the same as those of the first part and divided into three groups according to the terms of maintenance: group 1 (4°C), group 2 (37°C) and group 3 (75°C). The mixed samples were then put in glass cylinders with an internal diameter of 8 mm and a height of 10 mm and the samples of groups 1, 2 and 3 were kept at 4, 37 and 75 °C, respectively. At the end of each part, the primary and final setting times were measured by Gilmore needle. Data were analyzed by SPSS using Kruskal-Wallis test (p<0.05).

**Results:** The findings of this study showed a significant reduction of the primary and final setting time of MTA for the samples of both parts of the study with an increase in ambient temperature (p<0.05).

**Conclusion:** This study indicated that increased ambient temperature caused a reduction in the setting time of MTA.

## Introduction

Mineral trioxide aggregate (MTA) was first introduced in dentistry in 1993 as a filling material for the root apex. MTA has also been recommended for the pulp capping, pulpotomy, blocking the pores in the teeth with open apex, restoration of root perforations and canal filling [**1**].

MTA was first presented as derivatives of calcium oxide, silicate dioxide, and aluminum oxide that are converted to calcium silicate, dicalcium silicate and tricalcium aluminate compounds. Later, it was found that MTA, in addition to having bismuth oxide radiopaque factor, is very much similar to Portland cement. Depending on the type of commercial product, MTA compounds are different and are composed of 75% Portland cement, 20% bismuth oxide and 5% calcium sulfate dihydrate (Dentsply Tulsa) or 80% Portland cement and 20% bismuth oxide (MTA Angelus) [**2**].

Moreover, MTA contains slight amounts of SiO2, CaO, MgO, Al2O3, K2SO4 and Na2SO4 [**3**,**4**]. Studies have shown that MTA and Portland cement have similar antimicrobial activities [**6**] and biocompatibility [**7**] in some properties such as the improvement of periapical lesions [**5**]. MTA is available in white and grey forms. The white type, which is aesthetically superior, lacks tetracalcium aluminoferrite. The color difference is mainly due to lower amount of iron oxide in white MTA. The grey type of MTA is not usually used in cases where beauty is concerned [**4**].

Clinically, MTA has numerous advantages such as high sealing potential [**8**,**9**] good biocompatibility [**10**,**11**], acceleration of the amelioration of periapical lesions [**12**,**13**] and halting root inflammatory resorption [**14**]. In addition, the setting time of MTA is not influenced by the unfavorable effect of humidity or blood [**15**]. Although MTA is preferred in many dental treatments due to its numerous advantages, it has some deficiencies, including the handling problem and the long setting time [**16**]. Studies have shown that the setting time of MTA is longer than the common canal fillings such as amalgam, super-EBA, Intermediate Restorative Material (IRM) [**17**] and biodentine [**18**]. The longer setting time of root canal fillings is an unfavorable property because it facilitates cement leakage in periapical procedures [**19**]. Clinically, some conditions like reverse filling and sealing perforation require a faster setting time in order to prevent dissolution of the components of the filling material by blood or tissue fluids [**20**]. As a generally accepted advantage, canal filling materials need to be immediately hardened after the insertion into the canal cavity to minimize the contact time of the filling material with vital tissues [**18**].

Various studies have been conducted to overcome the longer setting time of MTA. Lee et al. reported that although adding hydration accelerators such as citric acid, lactic acid, sodium chloride, and calcium lactate gluconate improves the setting time, it reduces the compressive strength during the mixing stage of MTA [**21**]. AlAnenzi et al. showed that adding KY, CaCl2 or NaOC1 liquids to grey MTA improves the handling properties and reduces the setting time [**22**]. Recently, an attempt has been made to replace MTA by a calcium aluminosilicate cement called Quick-Set with a faster setting time. Although this material was similar to MTA in many restorative properties of periapical tissues, it resulted in more inflammation [**23**].

Aesthetically, white MTA is superior to the grey MTA [**4**] but the setting time of white MTA is longer than that of the grey type [**1**]. Further studies are needed to analyze the long setting time of MTA, especially the white type. This study investigated the effect of ambient temperature on the setting time of commercial white MTA.

## Materials and methods

In this experimental study, white MTA (Angelus, Brazil) was used. The effect of temperature was evaluated in two Parts.

In the first part, 9 packages of MTA (each containing 0.5 gr MTA) and 9 distilled water ampoules (5 ml) were divided into three groups (n=3) according to the ambient temperature:

- group one at 4°C (in refrigerator for 24 hours)

- group two at 37°C (in incubator for 24 hours)

- group three at 90°C (in incubator for 24 hours)

The samples were immediately mixed with 3/ 1 proportion (MTA powder to distilled water) according to the manufacturer’s instruction. The mixed materials were placed in glass cylinders (8 mm internal diameter and 10 mm height) and kept in incubator at 37°C and 100% humidity.

In the second part, the samples were prepared similarly to those of the first part and divided into three groups based on the maintenance conditions:

- group one at 4°C (in refrigerator for 24 hours)

- group two at 37°C (in incubator for 24 hours)

- group three at 75°C (in incubator for 24 hours)

Then, the samples were immediately mixed with 3/ 1 proportion (MTA powder to distilled water) according to the manufacturer’s instruction. The mixed materials were poured in glass cylinders (8 mm internal diameter and 10 mm height) and the samples of group 1, 2 and 3 were kept at 4, 37 and 75 °C, respectively under 100% humidity.

At the end of each part, the setting time of samples was measured by Gilmore needle according to similar studies [**24**-**27**]. Gilmore needle is composed of a metal frame on which two vertical axes are installed. A weight, to the end of which a needle with a specified diameter is attached, is installed on each of these vertical axes. A thin needle (415.6 gr) is attached to the bigger weight and a thick needle (2.12 mm in diameter) is attached to the smaller weight. The samples were tested at intervals of 1 or 5 minutes.

The primary and final setting times were respectively considered the times that the tip of light and heavy weights did not have an observable effect on MTA surface. The setting time of each sample was recorded. The obtained data were analyzed by SPSS software using Kruskal-Wallis test. P<0.05 was considered significant.

## Results

The setting time of MTA was analyzed by two primary and final needles at different temperatures in incubator and sterile liquid. As reported in **Table 1**, in the primary needle test, the temperature of 4°C indicated the highest mean value for the setting time of MTA and the temperature of 75°C showed the lowest setting time with the mean time of 20 minutes. Based on the results of Kruskal-Wallis test, this difference was reported to be significant (p<0.023). As for the final needle test, the temperature of 4°C with the mean time of 87 showed the highest mean value for the setting time of MTA and temperature of 75°C indicated the lowest mean value for the setting time of MTA. To analyze and confirm the difference between the effects of different temperatures on the setting time of MTA, Kruskal-Wallis test was run and the findings showed a significant difference between temperatures (p<0.025).

**Table 1** presents the number, mean values, and standard deviation of the setting time of MTA at different temperatures in incubator. According to **Table 2**, in the primary needle, the maximum mean setting time was reported for 4°C with the mean time of 43 minutes. Also, the minimum mean setting time was reported for 90°C with the mean time of 20 minutes. The results of Kruskal-Wallis test revealed that this difference was statistically significant (p<0.02). In the final needle test, the maximum mean setting time was observed at the temperature of 4°C with the mean time of 93 minutes. Further, the minimum mean setting time in the final needle test was reported for the temperature of 90°C with the mean time of 38 minutes. The findings of Kruskal-Wallis test showed this difference to be statistically significant (p<0.023). **Tables 2**-**1** show the number, mean value, and standard deviation of the setting time of MTA at different temperatures of sterile liquid.

**Table 1:** Number, mean value, and standard deviation for the setting time of MTA at different temperatures in incubator

		Primary needle			Final needle	
Temperature	Number	Mean	SD	Number	Mean	SD
4°C	3	42	3	3	87	3
37°C	3	27	3	3	47	3
75°C	3	20	0	3	37	3

**Table 2:** Number, mean value, and standard deviation for the setting time of MTA

		Primary needle			Final needle	
Temperature	Number	Mean	SD	Number	Mean	SD
4°C	3	43	3	3	93	3
37°C	3	25	3	3	75	3
90°C	3	20	0	3	38	3

## Discussion

One of the major problems of MTA is its long setting time. The current study investigated the effect of ambient temperature on the setting time of MTA. The results indicated a difference in the setting time at different temperatures. The results of the primary and final needle tests in incubator indicated that the minimum and maximum setting times of MTA were reported for the samples kept at temperatures of 4 and 75 °C, respectively. However, similar results were obtained for distilled water at different temperatures. These findings demonstrate that temperature can affect the setting time of MTA and the setting time reduces with a rise in temperature.

To confirm the effect of ambient temperature on MTA properties, Saghiri et al. showed that temperature could influence the surface hardness and the microscopic structure of MTA [**24**]. The effect of temperature on the setting time of MTA can be attributed to the chemical processes of the main material of MTA, Portland cement. Portland cement is hardened in contact with water; this is when a chemical reaction occurs between water and cement [**28**]. It has been shown that the size of particles [**29**], proportion of powder to liquid [**30**], presence of air in the mixture [**31**] and ambient temperature [**32**,**33**] can affect the physical properties, hydration process and synthesis of Portland cement. The hydration of MTA produces calcium hydroxide and hydrate silicate calcium (H-S-C) gel. H-S-C gel results in cement resistance, thereby contributing to the stability of hydrated cement [**34**]. According to the research, the hydration of Portland cement reacts to temperature and this reaction is pyrogenic [**35**,**36**].

In the present study, the setting time of MTA varied from 37 to 93 minutes depending on the ambient temperature or distilled water temperature. In previous studies, different setting times have been reported for the white MTA (ProRoot MTA), 165 ± 5 minutes [**23**], 170 ± 2 minutes [**37**] and 228.33 ± 2.88 [**18**], which are higher than those of the present study. Other studies have also reported a longer setting time for MTA than that of the current study [**38**-**43**], which can be due to the conditions of using distilled water regardless of temperature.

## Conclusion

The results of this study showed that ambient temperature before and after mixing has a significant impact on the setting time of MTA and the increased ambient temperature reduces the setting time.

## References

[1] Parirokh M, Torabinejad M (2010). Mineral trioxide aggregate: a comprehensive literature review-Part I: chemical, physical, and antibacterial properties. J Endod.

[2] Dorileo MC, Bandeca MC, Pedro FL, Volpato LE, Guedes OA, Dalla Villa R (2014). Analysis of metal contents in Portland Type V and MTA-based cements. Scientific World Journal.

[3] Asgary S, Shahabi M, Jafarzadeh T, Amini S, Kheirieh S (2008). The properties of a new endodontic material. J Endod..

[4] Patel N, Patel K, Baba SM, Jaiswal S, Venkataraghavan K, Jani M (2014). Comparing gray and white mineral trioxide aggregate as a repair material for furcation perforation: an in vitro dye extraction study. J Clin Diagn Res.

[5] da Silva SR, da Silva Neto JD, Veiga DF, Schnaider TB, Ferreira LM (2015). Portland cement versus MTA as a root-end filling material. A pilot study. Acta Cir Bras..

[6] Hasan Zarrabi M, Javidi M, Naderinasab M, Gharechahi M (2009). Comparative evaluation of antimicrobial activity of three cements: new endodontic cement (NEC), mineral trioxide aggregate (MTA) and Portland. J Oral Sci.

[7] Koçak S, Erten H, Baris E, Türk S, Alaçam T (2014). Evaluation of the biocompatibility of experimentally manufactured portland cement: An animal study. J Clin Exp Dent.

[8] Lee SJ, Monsef M, Torabinejad M (1993). Sealing ability of a mineral trioxide aggregate for repair of lateral root perforations. J Endod.

[9] Singh P, Paul J, Al-Khuraif AA, Vellappally S, Halawany HS, Hashim M, Abraham  NB, Jacob V, Thavarajah R (2013). Sealing ability of mineral trioxide aggregate, calcium phosphate cement, and glass ionomer cement in the repair of furcation perforations. Acta Medica (Hradec Kralove).

[10] Yoshimine JC, Ono M, Akamine A (2007). In vitro comparison of the biocompatibility of mineral trioxide aggregate, 4META/MMA-TBB resin, and intermediate restorative material as root-end-filling materials. J Endod.

[11] Chang KC, Chang CC, Huang YC, Chen MH, Lin FH, Lin CP (2014). Effect of tricalcium aluminate on the physicochemical properties, bioactivity, and biocompatibility of partially stabilized cements. PLoS One.

[12] Chang SW, Oh TS, Lee W, Cheung GS, Kim HC (2013). Long-term observation of the mineral trioxide aggregate extrusion into the periapical lesion: a case series. Int J Oral Sci..

[13] Paul ML, Mazumdar D, Vyavahare NK, Baranwal AK (2012). Healing of the periapical lesion in posterior teeth with mineral trioxide aggregate using orthograde technique - Two case reports. Contemp Clin Dent..

[14] Sharifi R, Parirokh M, Satvati SR, Torabinejad M (2014). Treatment of inflammatory root resorption using mineral trioxide aggregate: A case report. Dental Hypotheses.

[15] Naik RM, Pudakalkatti PS, Hattarki SA (2014). Can MTA be: Miracle trioxide aggregate?. J Indian Soc Periodontol.

[16] Butt N, Talwar PS, Chaudhry S, Nawal RR, Yadav S, Bali A (2014). Comparison of physical and mechanical properties of mineral trioxide aggregate and Biodentine. Indian J Dent Res.

[17] Torabinejad N, Hong CU, McDonald F, Pitt Ford  TR (1995). Physical and chemical properties of a new root-end filling material. J Endod.

[18] Kaup M, Schäfer E, Dammaschke T (2015). An in vitro study of different material properties of Biodentine compared to ProRoot MTA.. Head Face Med.

[19] Parirokh M, Torabinejad M (2010). Mineral trioxide aggregate: a comprehensive literature review-Part III: Clinical applications, drawbacks, and mechanism of action. J Endod..

[20] Bramante CM, Kato MM, Assis GF, Duarte MA, Bernardineli N, Moraes IG (2013). Biocompatibility and setting time of CPM-MTA and white Portland cement clinker with or without calcium sulfate. J Appl Oral Sci..

[21] Lee BN, Hwang YC, Jang JH, Chang HS, Hwang IN, Yang SY, Park YJ, Son HH, Oh WM (2011). Improvement of the properties of mineral trioxide aggregate by mixing with hydration accelerators. J Endod.

[22] AlAnezi AZ, Zhu Q, Wang YH, Safavi KE, Jiang J (2011). Effect of selected accelerants on setting time and biocompatibility of mineral trioxide aggregate (MTA). Oral Surg Oral Med Oral Pathol Oral Radiol Endod.

[23] Kohout GD, He J, Primus CM, Opperman LA, Woodmansey KF (2015). Comparison of Quick-Set and mineral trioxide aggregate root-end fillings for the regeneration of apical tissues in dogs. J Endod.

[24] Saghiri MA, Lotfi M, Joupari MD, Aeinehchi M, Saghiri AM (2010). Effects of storage temperature on surface hardness, microstructure, and phase formation of white mineral trioxide aggregate. J Endod.

[25] International Organization for Standardization Dental root canal sealing materials. ISO 6876:2001.

[26] (2013). ASTM C266-13, Standard Test Method for Time of Setting of Hydraulic-Cement Paste by Gillmore Needles, ASTM International. West Conshohocken, PA, www.astm.org.

[27] Wiltbank KB, Schwartz SA, Schindler WG (2007). Effect of selected accelerants on the physical properties of mineral trioxide aggregate and Portland cement. J Endod.

[28] Taylor HFW (1997). Cement chemistry.

[29] Osbaeck B, Johansen V (1989). Particle size distribution and rate of strength development of Portland cement. J Am Ceram Soc.

[30] Bentz DP (2006). Influence of water-to-cement ratio on hydration kinetics: simple models based on spatial considerations. Cem Concr Res.

[31] Buenfeld N, Okundi E (1999). Release of air from unsaturated aggregate during setting of concrete. J Construction and Building Materials.

[32] Klieger P (1958). Effect of mixing and curing temperature on concrete strength. ACI J Proc.

[33] Mouret M, Bascoul A, Escadeillas G (2003). Strength impairment of concrete mixed in hot weather: relation to porosity of bulk fresh concrete paste and maturity. Mag Concrete Res..

[34] Camilleri J (2007). Hydration mechanisms of mineral trioxide aggregate. Int Endod J.

[35] Escalante-Garcı´a JI, Sharp JH (2001). The microstructure and mechanical properties of blended cements hydrated at various temperatures. Cem Concr Res.

[36] Price WH (1951). Factors influencing concrete strength. J Aci J Proc..

[37] Gandolfi MG, Iacono F, Agee K, Siboni F, Tay F, Pashley DH, Prati C (2009). Setting time and expansion in different soaking media of experimental accelerated calcium-silicate cements and ProRoot MTA. Oral Surg Oral Med Oral Pathol Oral Radiol Endod.

[38] Dammaschke T, Gerth HU, Zuchner H, Schafer E (2005). Chemical and physical surface and bulk material characterization of white ProRoot MTA and two Portland cements. Dent Mater.

[39] Budig CG, Eleazer PD (2008). In vitro comparison of the setting of dry ProRoot MTA by moisture absorbed through the root. J Endod.

[40] Watts JD, Holt DM, Beeson TJ, Kirkpatrick TC, Rutledge RE (2007). Effects of pH and mixing agents on the temporal setting of tooth-colored and gray mineral trioxide aggregate. J Endod.

[41] Coomaraswamy KS, Lumley PJ, Hofmann MP (2007). Effect of bismuth oxide radioopacifier content on the material properties of an endodontic Portland cement-based (MTA-like) system. J Endod.

[42] Lee YL, Lee BS, Lin FH, Yun Lin A, Lan WH, Lin CP (2004). Effects of physiological environments on the hydration behavior of mineral trioxide aggregate. Biomaterials.

[43] Storm B, Eichmiller FC, Tordik PA, Goodell GG (2008). Setting expansion of gray and white mineral trioxide aggregate and Portland cement. J Endod.

